# Roles of miRNA dysregulation in the pathogenesis of multiple myeloma

**DOI:** 10.1038/s41417-020-00291-4

**Published:** 2021-01-05

**Authors:** Dan Chen, Xinhong Yang, Min Liu, Zhihua Zhang, Enhong Xing

**Affiliations:** 1 Department of Central Laboratory, The Affiliated Hospital of Chengde Medical College, Chengde, Hebei China; 2 Department of Hematology, The Affiliated Hospital of Chengde Medical College, Chengde, Hebei China

**Keywords:** Myeloma, Gene regulation

## Abstract

Multiple myeloma (MM) is a malignant disease of plasma cells with complex pathology, causing significant morbidity due to its end-organ destruction. The outcomes of patients with myeloma have significantly improved in the past couple of decades with the introduction of novel agents, such as proteasome inhibitors, immunomodulators, and monoclonal antibodies. However, MM remains incurable and presents considerable individual heterogeneity. MicroRNAs (miRNAs) are short, endogenous noncoding RNAs of 19–22 nucleotides that regulate gene expression at the posttranscriptional level. Numerous studies have shown that miRNA deregulation is closely related to MM pathology, including tumor initiation, progression, metastasis, prognosis, and drug response, which make the complicated miRNA network an attractive and marvelous area of investigation for novel anti-MM therapeutic approaches. Herein, we mainly summarized the current knowledge on the roles of miRNAs, which are of great significance in regulating pathological factors involved in MM progressions, such as bone marrow microenvironment, methylation, immune regulation, genomic instability, and drug resistance. Meanwhile, their potential as novel prognostic biomarkers and therapeutic targets was also discussed.

## Introduction

MM, characterized by the clonal expansion of monotypic plasma cells in the bone marrow and excessive production of monoclonal protein in the blood [1], is one of the most common hematological malignancies in adults worldwide [2]. Clinically, the symptoms of MM involve lytic bone disease, hypercalcemia, anemia, renal impairment, and immune paresis. Translocations (4; 14), (14; 16), 17p13 deletion, and 1q21 amplification are closely correlated with the pathogenesis of MM, and usually predict poor prognostic [3, 4]. Over the past two decades, strategies for MM therapy have been evolved rapidly, and the survival for patients with MM has also been significantly improved owing to their free access to effective therapeutic agents with acceptable toxicity, such as proteasome inhibitors, immunomodulators, and monoclonal antibodies [5]. Unfortunately, MM remains incurable, and almost all patients with MM still suffer relapsing even though they received the above novel agent-based therapy [2].

miRNAs are short, endogenous noncoding RNAs of 19–22 nucleotides. Emerging studies have revealed that miRNAs mainly regulate gene expression at the transcriptional level by binding to target mRNAs through their 3′ untranslated regions and recruiting the RNA-induced silencing complex, thereby leading to repression or promotion of DNA translation [6–8]. Presently, compelling findings have shown that depending on the complex regulatory network, miRNAs are capable of regulating a variety of biological processes, including cell differentiation, proliferation, apoptosis, autophagy, and stem cell maintenance. To date, significant findings have reported that a single miRNA could mediate more than 200 mRNAs, and approximately 50% of cell protein-encoding genes are regulatory controlled by miRNAs [9, 10].

In recent years, many miRNA expression abnormalities have been found related to pathogenesis and management in a variety of cancers, including MM. Some studies even tried to better clarify the critical pathogenesis of MM by analyzing and exploiting the role of miRNA. Unfortunately, these studies mainly focused on unilaterally exploring the interaction of miRNAs with MM, and whether/how miRNAs interact with MM in multiple fields or integrally is still not clear. Therefore, in this review, we will take advantage of the current understanding of miRNAs and discuss its role in regulating pathological factors involved in MM development, including regulatory of immunomodulation, tumor microenvironment, DNA methylation, genomic instability, and drug resistance, as well as acting as potential prognosis biomarkers and therapeutic targets.

## miRNA and MM

Enormous studies have revealed that miRNAs dysregulation is widely involved in the pathophysiological process of human malignancies. Further studies have shown that miRNAs are capable of regulating both carcinogenic pathways and tumor suppressor pathways in cancer. In addition, researchers even found that the expression of miRNAs themselves can be regulated by oncogenes or tumor suppressor genes [11].

Highly dependent on the cellular context, miRNAs are academically divided into oncogenic miRNAs and tumor suppressor miRNAs [12]. In general, the former specifically refers to miRNAs with increased expression in cancer cells and contributes to cancer development by inhibiting tumor suppressor genes, while the latter refers to miRNAs with downregulated expression in cancer cells and normally prevents cancer procession by suppressing the expression of proto-oncogenes [13].

Over the last two decades, a great effort has been employed to identify the abnormal expression of miRNAs in MM, in an attempt to select the optimal candidates to be investigated in MM therapy. In 2005, Masri A and his group firstly detected miRNAs expression in both MM patient samples and human myeloma cell lines (HMCL), and they observed that malignant plasma cells in both patient samples and cell lines presented significant differences in miRNA expression profiles compared with those of healthy subjects [14].

**Fig. 1:**
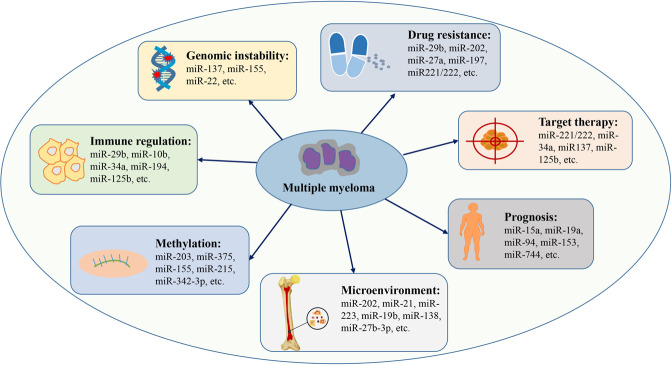
Important roles of miRNAs in the progression of multiple myeloma. MiRNAs are of great significance in regulating pathological factors involved in MM progressions, such as bone marrow microenvironment, methylation, immune regulation, genomic instability, and drug resistance. Meanwhile, miRNAs presented great potential as novel prognostic biomarkers and therapeutic targets.

Up to now, there are two types of studies, including Global miRNA expression investigations and functional verifications in vitro models with HMCL or in vivo mouse models, focusing on elucidating the role of miRNA in the pathogenesis of MM in an effort to illustrate the underlying mechanism [15]. Fortunately, numerous results, which are of great significance and prominence, have been discovered and summarized [15]. Here, we will mainly discuss the counteraction between miRNA and MM pathogenesis in the following aspects (as shown in Fig. 1).

### Involvement of miRNA in the bone marrow microenvironment

For years, researchers have paid their full attention to tumor cells and committed to deciphering their biological behavior, in an attempt to guide clinical cancer therapy. However, recent information regarding cancer biology has revealed that not only the tumor cell themselves but also the surrounded microenvironment played important roles in tumor oncogenesis, procession, metastasis, and relapse [16]. To survive and proliferate, MM cells have to dynamically interact with the bone marrow microenvironment [17, 18], which comprises feeder cells such as mesenchymal stem cells, stromal cells, fibroblasts, immune cells, and extracellular matrices. As revealed by extensive research, bone marrow stromal cells could protect MM cells against apoptotic signals, especially induced by drugs. This protective effect occurs mostly due to adhesion of MM cells to mesenchymal stem cells, thereby leading to cascade changes including secretion of cytokine and growth factors by latter cells (or both), activation of various genes and signaling pathways in both cell types, and induction of cell survival, proliferation and drug resistance in MM cells [19–21].

It has been widely recognized that miRNAs played important roles in the context of the bone marrow microenvironment by mediating their target genes, signaling molecules, and pathways, further affecting the pathogenesis of MM. In this regard, Shen et al. [22] demonstrated that miRNA-202 in bone marrow stromal cells can affect the growth and adhesion of MM cells by regulating B cell-activating factors. Wang et al. [23] detected the expression of miR-21 in MM cells adhered to bone marrow mesenchymal stem cells (BMSCs) and found that miR-21 was significantly upregulated. Leone et al. also demonstrated that HMCLs showed increased miR-21 expression when adhered to BMSCs. They found that inhibition of miR-21 significantly impaired cell viability and blocked clonogenic growth of MM cells in stroma-free conditions [24]. Reportedly, expression of some miRNAs, including miR-223, miR-16, miR-519d, and miR-485-5p were found highly expressed in MM-BMSCs compared to normal counterparts [25]. In addition, it was confirmed that upregulation of miR-29b in the bone marrow microenvironment can impair osteoclast differentiation and impede osteoclast activation induced by MM cells [26]. Conversely, Tsukamoto et al. [27] demonstrated that miR-138 presented high expression both in MSCs and MM cells, and inhibition of miR-138 can strengthen bone formation in the bone marrow niche. Besides, some miRNAs in BMSCs could be modulated after interaction with MM cells, resulting in angiogenesis induction [28] or osteogenesis impairment [29].

In addition, some studies have shown that miRNA can also play a regulatory role in the tumor microenvironment via being carried and secreted by exosomes. For example, Roccaro et al. [30] found miRNAs expression profiles in exosomes secreted by stromal cells derived from MM bone marrow were obviously different compared to exosomes secreted by normal stromal cells. Dependent on myeloma cells-derived exosomes, the expression of miR-214-3p and miR-27b-3p in bone marrow fibroblasts was found in step with MM progression and negatively regulate myeloma fibroblast apoptosis. Specifically, enforced expression of miR-214-3p and miR-27b-3p can promote proliferation and apoptosis resistance in myeloma fibroblasts, whereas suppression of them can reduce the expression of the antiapoptotic factor MCL1. Besides, researchers have found that overexpression of miR-146a in MSCs can enhance the secretion of IL-6, IL-8, IP-10, MCP-1, and CXCL1, thereby resulting in the improvement of MM cell viability and migration ability [31]. What is more, researchers have found that 19 kinds of miRNAs, including miR-146a, were dysregulated in MSCs when cultured with a conditioned medium for MM cells [31].

As a matter of fact, strengthening our knowledge of miRNAs’ roles in MM cell-bone marrow microenvironment interaction would be extremely important for us to find potential mechanisms that contribute to MM progression. However, at present, we still do not know what signals triggered miRNAs expression alterations in MM cell–bone marrow microenvironment context, which critical oncogenes or tumor suppressor genes are targeted by miRNAs in this context. Moreover, we so far do not know the possibility of involvement regarding other miRNAs, as well as their related potential targets and signaling pathways. Thus, more in-depth researches are warranted to better disclose the regulatory role of miRNAs in this context, so as to design more effective and attractive miRNA-based potential therapeutic tactics targeting MM cells in the context of their natural microenvironment.

### Regulation roles of miRNA in the immune response

At present, the emergence of immune therapies has become one of the great progress in the field of cancer therapy. Therapeutic strategies aimed to target the immune system have been widely applied to human malignancies including MM and shown promising outcomes both in pre-clinical models and clinical trials. Recently, miRNAs are becoming a hot spot in cancer research owing to their ability to regulate the immune system through various mechanisms, which include affecting the differentiation and activity of immune cells, reprogramming the molecular of immune cells, and modulating the secretion of inflammatory factors [32]. These characteristics, combined with the antitumor activities of miRNA synthetics (miRNA mimics or inhibitors), make the complicated miRNA network an attractive and marvelous area for exploring novel anti-MM therapeutic strategies.

Depending on the microenvironment cytokines and/or initial stimulus, Naïve T helper (Th) lymphocytes would differentiate into effector CD4+ cells, including Th1, Th2, Th17, and regulatory T (Treg) cells, which would later protect the host against diseases caused by a virus, intracellular bacteria or cancer cells [33–35]. Interestingly, miR-125b has been recently found to induce direct anti-MM activity both in vitro and in vivo by effectively targeting IRF4, an important molecular involved in the development and differentiation of Treg, Th2, Th9, and Th17 cells [36]. MiR-29b can directly regulate Th1 differentiation by targeting IFN-γ, thus presenting an important anti-myeloma activity [37]. In another study, the role of miR-21 in Th17-mediated MM tumor growth was investigated, in which researchers found that early inhibition of miR-21 in naive T cells can impair Th17 differentiation in vitro and abrogate Th17-mediated MM cell proliferation and osteoclasts activity [38].

Recently, authors discovered an IL-17/miR-192/IL17Rs regulatory feedback loop, in which activation of the IL-17 signal cascade induces NF-kB signaling and that, in turn, inhibits the miR-192 promoter. Besides, they found that miR-192 can directly target different IL-17 receptors [39]. A study evaluated the potential correlation between abnormal expression of miRNAs and Th17-associated cytokines in MM patients [40]. They found upregulated expression levels of IL-17, IL-21, IL-27, and downregulated expression levels of IL-22 in peripheral blood of MM patients than those in healthy donors. Meanwhile, these results were clearly connected with low expression of miR-15a/16, miR-34a, miR-194, and high expression of miR-181a in the bone marrow mononuclear cells of MM patients [40].

In addition, miRNAs could also play important roles in regulating the expression of ligands (such as MICA, MICB, and ULBP1), receptors, and cytotoxic enzyme involved in NK cell-induced antitumor activity [41]. Specifically, miR-10b overexpression in MM cells can inhibit MICB expression and impair their sensitivity to NK cell-mediated lysis. Accordingly, miR-10b antagonism extended NKG2D mediated killing of tumor cells both in vitro and in vivo [42].

Dendritic cells (DCs) cells have been described to play a pivotal role in the MM, such as infiltration (up to 10% of total cellularity) in the bone marrow of MM patients [43], maintaining MM cells growth [44], and protecting MM cells from melphalan- and bortezomib-induced apoptosis [14]. Of note, a growing number of studies have shown the important regulatory role of miRNAs in DCs function. For example, upregulation of miR-301a in DCs can inhibit IL-12, IL-6, and TNF-α secretion and mediate T-cell responses [45]. MiR-222, together with miR-155, was demonstrated to increase IL-12 secretion in DCs through the downregulation of SOCS1 [46]. Furthermore, miR-221 overexpression in DCs could promote the secretion of pro-inflammatory cytokines IL-6 and TNF-α [47], which may support MM cell growth. Lastly, recent studies have demonstrated that overexpression of miR-22 or miR-29a in DCs can impede their efficiency to drive a Th-17 response by reducing the expression of IL-6 and IL-23 [48, 49].

Besides, authors have observed an increased number of myeloid-derived suppressor cells (MDSCs) in peripheral blood and bone marrow extractions from MM patients, and they demonstrated that MDSCs are capable of differentiating into osteoclast-like cells, further leading to the MM-associated lytic bone disease, indicating a major role for MDSCs in MM pathobiology [18, 50, 51]. Experiment results showed that upregulated expression of miR-21 and miR-155 in MM cells can increase the number of MDSCs by directly targeting SHIP-1 and PTEN, respectively [52]. In addition, studies have found that miR-17-5p and miR-20a are able to impair the suppressive function of MDSCs by reducing their potential to block CD4 and CD8 T-cell responses [53, 54].

It has been reported that macrophages, an important component of stromal cells, play major roles in the biology of MM. In active MM (including relapse, and refractory period), macrophages can be recruited and activated by inflammatory factors including VEGF, FGF-2, and HGF [55]. In addition, macrophages in bone marrow can also protect MM cells from melphalan and spontaneous induced apoptosis [56]. MiR-155 was reported to promote the activation of macrophages thereby enhancing the pro-inflammatory response relying on the decreased expression of SOCS1, BCL6, and IL13RA [57–59]. MiR-125b also could promote the physiological activation of macrophages [60] and its expression would be quickly downregulated after inflammatory stimuli [61]. MiR-187 was reported to be involved in macrophage activation in an IL10 dependent manner, and high expression of miR-187 could inhibit LPS induced TNF-α, IL-6, and IL12p40 transcription, thus playing a significant role in inflammatory response suppression [62].

The above reports showed that miRNAs are closely involved and play important roles in immune regulation, further providing the theoretical basis for the exploitation of miRNAs as new therapeutic agents. However, we still have to fulfill our knowledge regarding the complex microenvironment during MM development and conduct appropriate clinical trials to identify the optimal and personalized therapeutic strategy, and as a result, achieveing improved outcomes for MM patients.

Unfortunately, there is still no documents reporting whether miRNAs were involved in MM pathologies by directly affecting the activity of DCs, MDSCs, and macrophages.

### Association between miRNA methylation and MM

It has been reported that several variations, including the abnormal activity of transcription factors, deregulation of miRNA host genes, and abnormalities in miRNA biogenesis pathways [63], could disrupt miRNA expression in MM. Besides these, epigenetic aberrations, especially the abnormal DNA methylation at miRNA promoter regions, seems to be one of the most important mechanisms in mediating aberrant miRNA deregulation [64–66]. The effects of epigenetic aberrations on gene expression are mainly reflected in the following aspects: the expression of genes with methylated CpG in the promoters was usually suppressed, while the expression of genes with un-methylated promoters was highly dependent on other regulation mechanisms [67, 68].

As a tumor suppressor, Wong et al. [69] have pointed out that the promoter region of miR-203 in MM is methylated, and the transfection of its precursor in MM cells can significantly inhibit their proliferation ability, thereby interfering with the progression of MM. Tatekawa et al. detected that miR-375 promoters were highly methylated both in HMCLs and patient-derived MM cells [70]. Misiewicz-Krzeminska et al. [71] found miR-155 was highly methylated in HMCLs, and high expression of miR-155 could improve the OS in MM. Wong et al. [72] reported that the promoter regions of the miR-124-1 were more frequently methylated in HMCLs than that in normal controls, and approximately 2% MM samples at diagnosis or relapse/progression also exhibited methylation in miR-124-1 promoter regions.

In spite of these, Pichiorri et al. demonstrated that the expression of miR-194-2-192 (11p13.1) cluster was reduced owing to DNA hypermethylation in HMCLs. They found that miR-194-2-192 cluster expression level could be upregulated by hypomethylation treatment. Moreover, they found that enforced expression of the miR-194-2-192 cluster can impair the proliferation activity and migration ability of MM cells [73].

In addition, reports have shown that the promoter regions of miR-34a and 34b/c were observed frequently methylated and their expression was inhibited in MM. Later, researchers found that their expression could be upregulated by the demethylating agent decitabine [74–76], and restoration of miR-34b could exert anti-MM activity in vitro [77]. The promoter region of miR-129-2 was also found hypermethylated in MM cells compared to normal cells, as well as in MM relapse/progression [78].

Moreover, the promoter regions of miR-342-3p, miR-10b-5p, and miR-152 were also observed methylated in MM cells compared to normal plasma cells, thus playing important roles in MM development [79, 80].

The above data fully demonstrated the important role of DNA methylation of miRNAs in the pathological process of MM, and further indicating that the corresponding MM tumor markers or molecular targeted drugs can be developed based on these results. However, we still do not know what factors induced miRNA promotor methylation and subsequent cascade changes, how to process it after methylation occurs to reduce adverse events, and whether there was other miRNAs methylation involved in the pathophysiological process of MM, etc. Thus, more in-depth researches are warranted to depict the complicated miRNAs methylation procedure in MM development.

### Roles of miRNA in regulating genomic instability in MM

Genomic instability is a characteristic of almost all cancers. It refers to events capable of causing alterations at both chromosomal and chromatin levels in a temporary or permanent manner [81]. Reports have shown that 25% of myeloma patients are characterized by genomic instability, which renders them resistant to chemotherapy and radiotherapy [82].

Of interest, recent evidence has implicated miRNA in the regulation of genomic instability, and therefore presents great potential in cancer therapy [83–85]. However, there are very few reports about miRNAs’ involvement in MM progression by regulating genomic instability pathways. Up to now, what we can learn is that in 2016, Qin and his group demonstrated that MM patients with low miR-137 expression had an increased frequency of IgH translocations t (4;14), while high miR-137 promoter methylation level was associated with t (4;14) IgH translocations. Also, they found that the ectopic expression of miR-137 decreased the incidence of chromosome 1q21 gains and 1p22.2, 14q, 17p13 deletions [86]. In addition, it is worth noting that Muvarak et al. [87] found that overexpression of miR-150 and -22 decreased the activity of an alternative form of the nonhomologous end-joining (ALT-NHEJ), leading to the impairment of DNA double-strand breaks repair and thereby, increasing the genomic instability in chronic myelogenous leukemia patients.

Considering that genomic instability identified in MM offers a unique therapeutic opportunity, therefore, while focusing on the role of miRNA in other aspects, researches on miRNA in this field should also be strengthened.

### Roles of miRNA in drug resistance

Due to the characteristics of MM disease, the application of chemotherapy drugs has been served as the main treatment strategy for many years and has been proven to be effective. However, the drug treatment itself has fallen into an awkward situation, that is, drug resistance. Indeed, drug resistance is the most critical and difficult obstacle in MM treatment, leading to the deterioration of MM disease, thus greatly hampering the survival and prognostic of MM patients. To solve this issue, studies have been focused on identifying mechanisms underlying drug resistance in MM, so as to develop novel and powerful therapeutic agents.

In recent years, the exploration of miRNAs in regulating the therapeutic effects of drugs has become a research focus in cancers, including MM [88, 89]. It was demonstrated that many miRNAs are involved in the modulation of sensitivity to anti-MM agents such as bortezomib, dexamethasone, and melphalan (as listed in Table 1). Among these, miR-29b can influence the bortezomib sensitivity to MM cells through the activation of a feedback loop with transcription factor Sp1 [37]. In addition, miR-202 can regulate MM cell’s sensitivity to bortezomib through downregulation of BAFF and JNK/SAPK signaling pathway [90]. Zhang et al. [91] showed that miRNAs in circulating exosomes, including miR-15a-5p, miR-16-5p, miR-17-5p, and miR-20a-5p were notably repressed in bortezomib-resistant MM patients compared to normal counterparts, indicating their important roles in bortezomib resistance. In addition, Ballabio et al. observed miR-27a expression was decreased in bortezomib-resistant MM cells than that in normal MM cells. They found ectopic expression of miR-27a can improve their sensitivity to bortezomib by inhibiting the expression of oncogene CDK5 [92].

**Table 1:** Correction between miRNA and related drug resistance in MM patients.

miRNA	Drug	Sensitivity	Target	References
miR-29b	Bortezomib	↑	Activating transcription factor Sp1	[37]
miR-202	Bortezomib	↑	Inhibiting the activation of JNK/SAPK signaling pathway	[82]
miR-15a-5p, miR-16-5p, miR-17-5p, and miR-20a-5p	Bortezomib	↑	Not mention	[83]
miR-27a	Bortezomib	↑	Inhibiting the expression of oncogene CDK5	[84]
miR-137 and miR-197	Bortezomib	↑	Inhibiting MCL-1 expression	[85]
miR-202	Bortezomib	↑	Inhibiting the activation of JNK/SAPK signaling pathway	[82]
miR-451	Bortezomib	↑	Inhibiting MDR1 expression	[94]
miR-202	Dexamethasone	↓	Inhibiting the activation of JNK/SAPK signaling pathway	[82]
miR-221/222	Dexamethasone	↑	Activating the ATG12/p27-mTOR autophagy-regulatory axis	[86]
miR-193a	Dexamethasone	↑	Inhibiting MCL-1 expression	[87]
miR-125b	Dexamethasone	↓	Inhibiting p53/miR-34a/SIRT1 signaling network	[88]
miR-137	Dexamethasone	↑	Inhibiting c-MET expression and AKT phosphorylation	[89]
miR-221/222	Melphalan	↑	Upregulation of the PUMA and modulation of SLC7A5/LAT1 and ABCC1/MRP1	[90]
miR-140-5p	Melphalan	↑	Inhibiting ATG14 expression	[91]
miR-451	Melphalan	↑	Inhibiting MDR1 expression	[93]
miR-152	Melphalan	↑	Inhibiting DKK-1 expression	[94]
miR-451	As_2_O_3_	↑	Inhibiting MDR1 expression	[93]
miR-202	Thalidomide	↓	Inhibiting the activation of JNK/SAPK signaling pathway	[82]

In a recently published work, Yang et al. found the expression of miR-137 and miR-197 was suppressed in MM cell lines and MM patients’ tumor cells, respectively. Further research showed that inhibition of such miRNAs can increase the viability of MM cells and reduce their sensitivity to bortezomib [93]. Another study conducted by Shen et al. [90] showed that upregulation of miR-202 improved the bortezomib sensitivity but reduced dexamethasone and thalidomide sensitivity to MM cells through targeting the JNK/SAPK signaling pathway.

As for dexamethasone, miR-221/222 inhibition has been found to significantly abrogate dexamethasone resistance in MM cells by targeting the ATG12/p27-mTOR autophagy-regulatory axis [94]. Wu et al. [95] demonstrated the expression levels of miR-193a were inversely correlated with dexamethasone sensitivity of MM cells. Besides, Murray et al. [96] reported that miR-125b could impair the sensitivity of MM cells to dexamethasone, thereby alleviating dexamethasone-induced cell death in MM cells. Moreover, Zhang et al. [97] found miR-137 improved the dexamethasone sensitivity to MM cells by weakening the c-MET expression and blocking the AKT phosphorylation.

When it comes to melphalan, Gulla et al. [98] showed that miR-221/222 repression could significantly rescue melphalan-sensitivity of MM cells through upregulation of the PUMA and modulation of SLC7A5/LAT1 and ABCC1/MRP1. Lu et al. [99] observed that miR-140-5p was down expressed in melphalan-resistant MM cells compared to normal counterparts and upregulation of miR-140-5p could suppress autophagy and resistance of melphalan-resistant MM cells. Viziteu et al. [100] showed that exogenous high expression of RECQ1 can protect MM cells from bortezomib- and melphalan-induced cytotoxicity, which was closely related to aberrant miR-203 deregulation. Also, Du et al. [101] found inhibition of miR-451 enhanced the sensitivity of MM cells to bortezomib, As_2_O_3_, and melphalan through reducing MDR1 expression. While Xu et al. [102] documented that miR-152 promoted the melphalan sensitivity of MM cells, and they found that application of melphalan could induce more cell death of MM cells in the presence of miR-152 mimic.

Although enormous miRNA studies in MM have been focused on revealing their role in MM-related drug resistance, only a few studies addressed the possible mechanisms, for example, p53-related signaling pathway [96, 103, 104], NF-κB signaling pathways [23], JNK/SAPK signaling pathway [90], chromosomal deletions [105, 106], as well as by regulating cell cycle, proliferation or survival [107–109]. Even though there are some studies confirming that the above signaling pathways or forms are involved, it is still not enough to clearly reveal the complicated miRNA network in mediating drug resistance, which will need a long way to go.

Besides, other immunomodulatory drugs, such as lenalidomide, pomalidomide, and carfilzomib, have also been regarded as a chemotherapy strategy for MM treatment, however, whether miRNAs are involved, by which forms or signaling pathways, remains unclear and needs to be further studied.

Nevertheless, it is necessary to mention that only a very small part of cells in the above studies are derived from primary MM cells of MM patients resistant to drugs or at relapse. In addition, these studies did not construct an appropriate in vitro drug resistance model of MM cells based on the primary MM cells from MM patients [110]. However, such issues were extremely important as we could directly get more relevant information regarding miRNAs contribution to MM drug resistance.

### Target agents of miRNA in MM therapy

In light of the fact that miRNAs are abnormal expressed in cancers and present oncogenic or tumor-suppressive properties by targeting multiple signaling pathways, the current era is witnessing miRNAs being exploited as new tumor therapy targets. Combined with the current understanding of miRNA and technical conditions, there is currently two effective and pronged approach for anti-cancer therapeutic strategies: inhibition of oncogenic miRNAs and replacement of tumor suppressor miRNAs. The former relates to inhibiting miRNAs that are highly expressed and promote survival of the cancer cells by using RNA molecules complementary (antagomirs), while the latter means restoring the function of tumor-suppressive miRNAs that are downregulated by using vector overexpressing a specific miRNA or by transient transfection of miRNA mimics. In many cases, the purpose of the above two strategies was to disturb the oncogenic properties of the cancer cells by impeding uncontrolled proliferation activity and inducing apoptotic cascade events.

There are few reports on the first strategy, which is to artificially inhibit the expression of miRNA in MM to hinder cancer cell progression. MiR-221/222 has been approved as a good candidate in MM target therapy. Artificial transfer of miR-221/222 inhibitors in MM cells triggered the up-regulation of their target genes, including PTEN, PUMA, p27Kip1, and p57Kip2, thereby exhibited in vitro antiproliferative effects in MM cells. Conversely, upregulation of miR-221/222 in MM cells increased the percentage of S-phase and redressed p27Kip1 expression, which is advantageous to MM progression [98, 111]. Credibly, more oncogenic miRNAs and their role in the progress of MM will be revealed in future research, thereby be applied to the development of targeted therapies on this basis.

However, there are many documents exploiting the second strategy for targeted therapy in MM, showing promising and exciting results. Di Martino et al. [112] have shown that transfection of miR-34a mimics or lentivirus overexpressing miR-34a in MM cells impaired proliferation and induced apoptosis of MM cells by suppressing the expression of CDK6, BCL-2, and NOTCH1. Yang et al. have found that the expression of miR-137/197 was significantly decreased both in MM cell lines and MM patient samples in contrast to their normal counterparts. Overexpression of miR-137/197 by transfection of synthetic mimics induced apoptosis and inhibited the proliferation, colony formation, and migration ability of MM cells via targeting MCL-1 [93]. Xu et al. detected that miR-1271 was reduced in primary MM cells, and they revealed that enforced expression of miR-1271 can block proliferation and trigger apoptosis of MM cells. As a comparison, the downregulation of miR-1271 exhibited the opposite effect [113]. Moreover, Morelli et al. [36] found that miR-125b is low expressed in MM cells, and overexpression of miR-125b by transfecting with lentiviral vectors or synthetic mimics reduced the proliferation and survival, as well as prompted apoptotic and cell death of MM cells via directly targeting IRF4 and BLIMP-1.

Other miRNAs are also observed downregulated in both MM cell lines and newly diagnosed MM patients while overexpression of them impeded cell proliferation, cycle evolution, and prompted apoptosis in MM, such as miR-29b [114], miR-15 and-16 [115], miR-152 [102], miR-324-5p [116], miR-338-3p [117], miR-19b [118], miR-129 [119], and so on.

It is important to point out that although studies demonstrated here suggest miRNAs as promising agents in MM target therapy, no miRNA molecule so far has been designed and conducted in MM patients’ clinical trials. The following reasons may explain this dilemma: firstly, how to maintain their stability in vivo, since they may be removed by endonucleases or by phagocytic cells. Secondly, how to efficiently deliver them to target cells. To date, several attempts have been exploited to overcome these challenges, including the conjunction of tumor-suppressive miRNAs with liposome nanoparticles, atelocollagen, and adenoviruses system [120]. In addition, considering that exosomes can be secreted by most cells in the body and have good stability, another effective method may become a better choice: combing oligonucleotides with targeting peptide engineered exosomes, so as to greatly increases the possibility of transporting such particular molecules to target cells or tissues.

### Significance of miRNA in the prognosis of MM patients

In the last two decades, a great number of studies have been focusing on exploring reliable prognostic biomarkers in cancer so as to guide clinical treatment, thereby improving outcomes of cancer patients [121, 122]. By evaluating the results of published literature, it was suggested that microRNA profiles could serve as such molecular biomarkers for multiple myeloma owing to different miRNAs have been found to be associated with different disease stage, thereby providing insights on the prognosis of MM patients [123].

In general, many miRNAs have been reported to be closely related to the prognosis of MM patients (as listed in Table 2). For example, miRNA-15a was found down expressed in MM cells and displayed prognostic significance in MM. Patients with low expression of miR-15a were accompanied with dramatically shorter progression-free survival (PFS) and overall survival (OS) [124]. Corthals S et al. observed that MM patients with high expression of the miR-194 have superior OS [125]. Yang et al. [126] demonstrated that miR-410 was closely associated with the ISS stage of MM patients, and patients with high expression of miR-410 have poorer OS and PFS. Hao et al. found that downregulation of miR-19a resulted in significantly decreased PFS and OS in MM patients [127]. In addition, Chi et al. [128] showed that low expression of several miRNAs, including miR-153, miR-296, miR-490, miR-455, miR-500, and miR-642 are corrected with superior event-free survival in MM patients, whereas high expression of miR-373, miR-548d, miR-554, and miR-888 predicted poorer prognosis. By analyzing miRNA expression profiles of 163 clinical samples from MM patients, Wu et al. [129] observed that patients with higher expression of miR-17 and miR-886-5p have shorter OS.

**Table 2:** Correction between miRNA expression and related prognostic significance in MM patients.

miRNA	Expression pattern	Prognostic significance	References
miR-15a	Downregulated	Poorer PFS and OS	[116]
miR-194	Upregulated	Superior OS	[117]
miR-410	Upregulated	Poorer PFS and OS	[118]
miR-19a	Downregulated	Poorer PFS and OS	[119]
miR-153	Downregulated	Superior event-free survival	[120]
miR-296	Downregulated	Superior event-free survival	[120]
miR-490	Downregulated	Superior event-free survival	[120]
miR-455	Downregulated	Superior event-free survival	[120]
miR-500	Downregulated	Superior event-free survival	[116]
miR-642	Downregulated	Superior event-free survival	[120]
miR-373	Upregulated	Poorer PFS and OS	[120]
miR-548d	Upregulated	Poorer PFS and OS	[120]
miR-554	Upregulated	Poorer PFS and OS	[120]
miR-888	Upregulated	Poorer PFS and OS	[120]
miR-17	Upregulated	Poorer PFS and OS	[121]
miR-886-5p	Upregulated	Poorer OS	[121]
miR-20	Upregulated	Poorer PFS	[122]
miR-92	Upregulated	Poorer PFS	[122]
miR-92a	Upregulated	Poorer PFS	[123]
miR-16	Downregulated	Poorer PFS and OS	[123]
miR-19b	Downregulated	Poorer PFS and OS	[123]
miR-25	Downregulated	Poorer PFS and OS	[123]
miR-744	Downregulated	Poorer PFS and OS	[123]
Let-7e	Downregulated	Poorer OS and TTP	[123]

Along the same line, by screening 85 newly diagnosed MM patients, miRNA-17, miR-20, and miR-92 were found up-regulated in those patients experiencing a shorter PFS [130]. What is more, results from a large analysis containing 1214 cases revealed that MM patients with high expression of miR-92a or low expression of miR-15a, miR-16, miR-19b, miR-25, miR-744, and let-7e have a poor prognosis [131].

Nevertheless, none of the above miRNAs so far has been definitively validated and identified as prognostic biomarkers in clinical practice. In addition, single studies themselves may have design flaws, thus unconvinced to achieve reliable and comprehensive results. Therefore, it is imperative to execute miRNA profiling using high-throughput next-generation sequencing to identify variations of these miRNAs and subsequently combined with exhaustive meta-analysis [132], together with advanced preclinical studies as previously described [133, 134], to construct multiparametric prognostic models for MM patients.

### Conclusion and future prospects

Since researchers first discovered miRNAs in the nematode *Caenorhabditis elegans* almost 20 years ago [135], studies focused on miRNAs have expanded swiftly and remarkably, leading to our comprehensively understanding of their great significance in regulating cell biology and cancer pathogenesis. Later, with the development of miRNA microarrays technology and high-throughput sequencing, researchers are interested in exploring how miRNA profiles are connected with tumor classification, deterioration, relapse, and prognosis prediction. Fortunately, they found that compared to mRNA expression profiles, miRNA profiles are more accurate and meaningful in distinguishing normal tissues from cancerous tissues, identifying the organ or tissue from which cancer cell originates, and even dividing subtypes of cancer cells [136].

The review here described the deregulation of miRNAs directly associated with pathological factors that contribute to MM progression, such as bone marrow microenvironment, DNA methylation, immune regulation, genomic instability, and drug resistance. In addition, we also summarized the current miRNAs which are capable of acting as therapeutic targets and prognostic biomarkers of MM patients. Based on the comprehensive and profound understanding of the roles played by miRNA networks in the pathophysiological process of MM, our ultimate goal is to identify the high-risk groups who are susceptible to MM, then exploit individualized optimal therapy to delay the deterioration and improve the clinical outcomes, so as to lengthen the PFS and OS in MM patients.

However, it must be mentioned that the transformation from preliminary basic studies to the clinical application regarding miRNAs acting as diagnostic markers and potential therapeutic targets remains in its infancy and faces the following challenges: (1) The pathological mechanism and metabolic regulation of tumor formation in animal models are significantly different from those in humans. It remains unclear whether the abnormal expression profiles of miRNA in animal models and the corresponding long-term effects are similar to those in humans; (2) for the collection and storage of different samples (such as tissue, plasma, urine, and saliva), extraction and purification of small RNA, and subsequent data processing and analysis, there is currently no unified standardized protocol. In addition, for the same sample, the results obtained from different high-throughput platforms or miRNA arrays may be different, so we are not able to confirm its initially and reliability; (3) In the complex network composed of miRNAs and their target genes, we cannot ensure that the selected therapeutic miRNAs will not affect other nonpathogenic genes, thus causing the unknown characteristics of long-term side effects caused by this application; (4) due to the particularity of the delivery system, how to achieve the highest safety and effectiveness of the delivery of miRNA vectors, and to ensure that miRNAs are not lysed before they exert therapeutic effects, are also problems that need to be solved urgently.
